# Collective action for implementation: a realist evaluation of organisational collaboration in healthcare

**DOI:** 10.1186/s13012-016-0380-z

**Published:** 2016-02-09

**Authors:** Jo Rycroft-Malone, Christopher R Burton, Joyce Wilkinson, Gill Harvey, Brendan McCormack, Richard Baker, Sue Dopson, Ian D. Graham, Sophie Staniszewska, Carl Thompson, Steven Ariss, Lucy Melville-Richards, Lynne Williams

**Affiliations:** 1 School of Healthcare Sciences, Bangor University, Bangor, UK; 2 School of Health Sciences, University of Stirling, Stirling, UK; 3 Alliance Manchester Business School, University of Manchester, Manchester, UK; 4 School of Nursing, University of Adelaide, Adelaide, Australia; 5 Division of Nursing, School of Health Sciences, Queen Margaret University, Musselburgh, UK; 6 Department of Health Sciences, University of Leicester, Leicester, UK; 7 Said Business School, University of Oxford, Oxford, UK; 8 Epidemiology and Community Medicine, University of Ottawa, Ottawa, Canada; 9 Royal College of Nursing Research Institute, University of Warwick, Warwick, UK; 10 School of Healthcare, Faculty of Medicine and Health, University of Leeds, Leeds, UK; 11 ScHARR, University of Sheffield, Sheffield, UK

**Keywords:** Implementation, Collaboration, Evidence, Co-production, Knowledge, Realist

## Abstract

**Background:**

Increasingly, it is being suggested that translational gaps might be eradicated or narrowed by bringing research users and producers closer together, a theory that is largely untested. This paper reports a national study to fill a gap in the evidence about the conditions, processes and outcomes related to collaboration and implementation.

**Methods:**

A longitudinal realist evaluation using multiple qualitative methods case studies was conducted with three Collaborations for Leadership in Applied Health Research in Care (England). Data were collected over four rounds of theory development, refinement and testing. Over 200 participants were involved in semi-structured interviews, non-participant observations of events and meetings, and stakeholder engagement. A combined inductive and deductive data analysis process was focused on proposition refinement and testing iteratively over data collection rounds.

**Results:**

The quality of existing relationships between higher education and local health service, and views about whether implementation was a collaborative act, created a path dependency. Where implementation was perceived to be removed from service and there was a lack of organisational connections, this resulted in a focus on knowledge production and transfer, rather than co-production. The collaborations’ architectures were counterproductive because they did not facilitate connectivity and had emphasised professional and epistemic boundaries. More distributed leadership was associated with greater potential for engagement. The creation of boundary spanning roles was the most visible investment in implementation, and credible individuals in these roles resulted in cross-boundary work, in facilitation and in direct impacts. The academic-practice divide played out strongly as a context for motivation to engage, in that ‘what’s in it for me’ resulted in variable levels of engagement along a co-operation-collaboration continuum. Learning within and across collaborations was patchy depending on attention to evaluation.

**Conclusions:**

These collaborations did not emerge from a vacuum, and they needed time to learn and develop. Their life cycle started with their position on collaboration, knowledge and implementation. More impactful attempts at collective action in implementation might be determined by the deliberate alignment of a number of features, including foundational relationships, vision, values, structures and processes and views about the nature of the collaboration *and* implementation.

## Background

The gap between evidence and practice has often been defined as a practice or service problem rather than one of knowledge creation [1]. This perspective is perpetuated by a ‘two communities’ [2] model of knowledge production in which the producers and users of research occupy separate worlds. Increasingly, it is recognised that such translational gaps might be narrowed by bringing the users and producers of research closer together. As such, initiatives increasingly focus on demonstrating the potential of collaboration. However, little empirical evidence about the conditions, processes or outcomes related to collaboration and evidence use exists. In this paper, we fill this gap by presenting an explanatory theory about collective action in implementation derived from a national longitudinal empirical study of collaboration.

### Framing the knowledge use challenge

Over time, there has been a shift (in the literature at least) from seeing knowledge and its use in practice as discrete events to conceptualising them as a process. Alongside this has developed a recognition that such processes are not necessarily linear, but that knowledge use is often multi-factorial and less predictable than is sometimes implied. Frameworks and theories have become increasingly focused on action in context [3–6]. The discourse used to describe this shift, reflecting underlying epistemologies, has moved from the linear (e.g. ‘research into practice’, ‘knowledge transfer’) to a more dynamic (e.g. ‘knowledge translation’, ‘knowledge mobilisation’, ‘engaged scholarship’) emphasis.

Practice-based, collaborative and organisational approaches to knowledge and its use are increasingly emphasised in contrast to an evidence-orientated view of knowledge use, which implies evidence as a ‘product’ needing to be pushed out to its users over an academic-practice boundary from one community to another [7–9]. Pushing out evidence as guidelines has had some, but relatively limited, success (given the investment in such products) in improving health outcomes. In theory, collaborations could blur this academic-practice boundary and the evidence would be co-produced within communities of practice, increasing the relevance to that community and its potential use [2]. It is this conceptualisation of implementation that we adopted in this study.

### Collaboration and the ‘knowing-doing’ gap

A variety of organisational collaborations between academia and practice have emerged over the past decade (e.g. the United States’ Quality Enhancement Research Initiative, Dutch Academic Collaborative Centres for Public Health, Australian Advanced Health Research and Translation Centres and England’s Academic Health Science Networks and Collaborations for Leadership in Applied Health Research and Care). Whilst collaboration between academia and services is often perceived as an effective means of closing the gap between knowing and doing, the causal pathway from developing partnerships, to the use of evidence in practice, and subsequent translation into improved patient outcomes is yet to be established.

A search of the literature (1994–2014) to answer the question, ‘why and how does organisational collaboration between researchers and practitioners enable implementation of evidence within a health service context’ [10], revealed 10 relevant papers. However, we found little evidence that *directly* linked collaboration to knowledge use, but they did reveal the features of collaborations likely to make them more successful. These included the following:

- Attention to communication mechanisms [11, 12],
- Setting intermediate outcomes/goals [13–15],
- Time and space need to be given to develop and implement plans [16, 17],
- Choice of topic with resonance and relevance [11],
- Closer physical proximity between partners [11],
- Re-balancing and sharing power [18, 19] (and allowing time to develop mutual trust and respect [18]).

It is unclear how and whether these collaborative conditions impact on knowledge use itself. Additionally, the existing evidence base is limited by a focus on researching one-off projects and/or events rather than on longitudinal and larger-scale organisational initiatives; consequently, it is not possible to speculate on the sustainability of such initiatives.

### Collaborations for Leadership in Applied Health Research & Care

Collaborations for Leadership in Applied Health Research & Care (CLAHRCs) were established in the English NHS in 2008 as collaborations between healthcare services and higher education organisations. Nine collaborations were funded as pilots by the National Institute for Health Research (NIHR) with approximately £100 million funding over 5 years with a further £100 million in ‘matched’ funds coming from the NHS. CLAHRCs were established following recommendations from a working group charged with developing an action plan for more effective and efficient health care in England [20, 21]. Each CLAHRC was funded to deliver three interlinked functions: conduct high quality applied health research, implement research into practice and increase capacity to engage with and apply research [22]. The implicit ‘theory’ was that providing a resource and structure would enable the research and practice community to work together to accelerate the implementation of research. We set out to investigate this theory, and fill a gap in the evidence about collaborative approaches to bridging translational gaps.

## Methods

### Approach

This study was a longitudinal realist evaluation using multiple-qualitative-methods case studies conducted between 2009 and 2014 [23]. Our purpose was to develop an explanatory theory about implementing research through CLAHRCs as collaborative entities and answer the realist question, ‘what works, for whom, how, why and in what circumstances?’

Realist evaluation is particularly appropriate for developing explanations about how programmes, which by their nature are complex, work contingently within the context of implementation [23–26]. We followed the realist approach offered by Pawson and Tilley [24]. Each CLAHRC’s approach(s) to implementation (the ‘programme’) was examined to identify what is it about them that ‘works’: their mechanisms and the contextual conditions that lead to outcomes. According to Pawson and Tilley [24], mechanisms are underlying causal forces that are usually unobservable and involve the reasoning of participants (of the programme/intervention—i.e. CLAHRC), which fire in particular contexts. Therefore, realist inquiry aims to uncover what it is about the context that affects whether or not mechanisms fire to produce outcomes. This configuration is commonly expressed as C + M → O. The study was designed and conducted with participants from CLAHRCs on the research team.

### Data collection

We focused on three CLAHRCs as case studies [27]. Sites were selected based on funders’ requirements for coverage alongside a need to manage burden (this was one of four projects researching nine CLAHRCs) and a CLAHRC’s willingness to engage in project design and development. We use the pseudonyms Oakdown, Ashrove and Hazeldean for the three cases. Consistent with a realist evaluation cycle, we conducted the study through phases of theory generation, theory testing and refining and programme theory specification [23, 26].

#### Approach

Realist evaluation is theory driven. Theory ‘tells us where to look’ and ‘what to look for…directs us to vital explanatory components…their inter-relationships and the things that bring about those interrelationships’ [25, p. 62]. The starting point for this study was the development of programme theory, which drove our data collection and analysis strategy [26]. We built a conceptual framework that helped to focus the first round of data collection (see [26] for fuller details of the framework’s development) around some initial hypotheses (Table 1). Following the analysis of round 1 data, the initial set of hypotheses was developed into context-mechanism-outcome formulae (CMOs), data collection was then focused on refining and finally, in round 4, testing this programme theory.

**Table 1:** Initial hypotheses (see [26] for more detail about how these hypotheses were developed, including a more in-depth consideration of their content)

The contexts of CLAHRCs will determine how the ‘programme’ plays out and will provide an explanation of those contexts that might be more appropriate or conducive. All action occurs within a context, which is multi-layered and multi-faceted. There is a growing evidence base about factors that have been identified that might explain whether contexts are more or less facilitative of implementation, including culture, communication, resources, leadership and tailoring of approaches/strategies (or not) to implementation contexts.
The way in which CLAHRCs’ interpret ‘knowledge’ will determine the importance and value they assign to different sources of knowledge and how these are privileged. Propositional and non-propositional source of knowledge have the potential to impact practice. Types of evidence from these sources (e.g. research, experience etc.) are often valued, and therefore privileged differently by different stakeholders.
How CLAHRCs develop ‘facilitation’ roles, including how they fit into their overall framework(s) for implementation, and the strategies, approaches and interventions they might employ will determine their success at supporting implementation-related activity. Facilitation and facilitators enable or make things easier—there are many roles that might (in theory) fulfil this function with a CLAHRC.
CLAHRCs with more effective patient and public involvement (PPI) strategies will achieve more relevant and impactful implementation. There is a very limited evidence base about PPI in implementation, but given what we know from PPI in research, for example through INVOLVE (http://www.invo.org.uk/), more relevant and impactful implementation may be determined by how they engage with stakeholder such as the public and patients in the locale.
How knowledge is prioritised and then particularised will vary within and across contexts, over time, and be prompted by the different choices of many stakeholders. How organisations store, share and learn from knowledge provides one indication of their capability as learning organisations. In theory, learning organisations are environments in which implementation and improvement might be more successful.
The way in which CLAHRCs’ respond to their local health, human and social geography will determine their ability to address implementation challenges that are important to the region. The CLAHRC’s commissioning brief was focused on delivering improvement in response to regional health priorities. Therefore, geography is an important aspect of a CLAHRC’s context, in that it has the potential to drive, shape and be impacted by service change.
How agents (those involved in producing and implementing CLAHRC work), beneficiaries (those that might profit/benefit from CLAHRC) and victims (those excluded or suffer opportunity costs) respond to the opportunities the CLAHRC offers, will help explain how and why the CLAHRC programme works (or not). As an interactive and deliberative endeavour, implementation processes and impacts are dependent on the individual and collective action of actors and agents working at different levels and places within the organisation(s).
A CLAHRC’s history, age and stage of development will impact on their approach and ability to implement knowledge. The funder’s expectation was for CLAHRCs to implement their own research within 3–5 years (this did not preclude them implementing existing research), placing an importance on the concept of time. Time therefore sets a frame of reference for any changes instigated, occurring and explained.
A CLAHRC’s approach to developing their formal and informal structures will vary and therefore will provide some insight into architectures that are more or less helpful for implementation through collaboration. In theory, structures and processes that enable closer engagement between health services and higher education should be those that facilitate relationship building and collaborative working.

Data were collected over four rounds and included the following:

*Semi-structured interviews*: with individual participants by telephone or face-to-face and guided by an interview spine. As we were developing explanations over time, the focus and therefore schedule for interviews reflected these iterations. The first round of data collection was exploratory and focused on the initial hypotheses; following analysis of round 1 data, data collected in rounds 2 and 3 were focused on helping us refine the emerging explanations. Interviews in round 4 were focused on ‘testing’ the explanations. Interviews were audio-recorded and lasted between 30 and 90 min.

*Observations*: Non-participant observation of some events and meetings (e.g. board meetings and stakeholder events) was conducted and recorded as field notes using nine dimensions of observation as a guide: space, actors, activities, objects, acts, events, time, goals and feelings [28].

*Documents*: A range of documents were gathered from each CLAHRC including newsletters, progress reports, job descriptions and implementation outputs—e.g. specific assessment tools, publications and outcomes—to help contextualise and complement other data sources.

*Stakeholder engagement*: We involved patients and the public through a stakeholder group that was set up for the project, and the wider CLAHRC community through attendance and presentations at their joint meetings. In the Interpretive Forum, we engaged policy makers, academics and the CLAHRCs as an opportunity to verify our emerging findings.

#### Sample

For each round of data analysis, the sampling framework was based on a stakeholder analysis [29] and used both theoretical and criterion sampling that determined which stakeholders were ‘essential’, ‘important’ and/or ‘necessary’ to involve [30]. Details of potential participants were provided by CLAHRC Directors or Programme Managers. Individuals were then invited to participate and given at least 24 h to consider their consent.

### Data analysis

Data analysis was iterative in order to build explanations over time and enable us to focus subsequent data collection in areas of productive enquiry. We used a combined inductive and deductive approach to ensure that the process continually focused on the propositional-building function of the CMO [25].

**Fig. 1:**
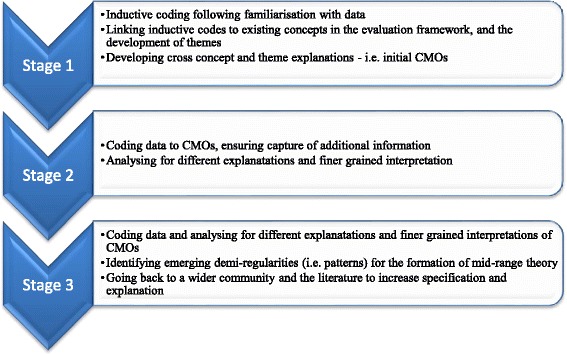
Stages of analysis

Following each round of data collection, analysis began with reading and re-reading the transcripts and field notes before coding. Consistent with case study methods, data were analysed within data sources, cases and then explanations developed across cases, with attention to the realist task of uncovering contingencies and conditions—i.e. the relationships between factors that explained CLAHRCs’ approach to implementation and the conditions in which they operated. We used interview data as our starting point and then moved on to observations and documents to help build explanations. In this sense, they were purposively mined for information that would help us refine/challenge/develop context-mechanism-outcome configurations and as such each stage of analysis (summarised in Fig. 1) became progressively focused. Practically, this was enabled through the use of mapping and charting both figuratively and through matrices. The analysis process was managed by three members of the core research team with regular engagement with members of the wider team, including those from participating CLAHRCs for sense checking.

We closed the realist evaluation loop by testing emerging findings with a wider community. An interpretive forum held in April 2014 was an opportunity to reflect on, interpret and surface viewpoints with a different group of stakeholders.

### Ethics

Ethical approval for the study was given from a multi-site research ethics committee (11/YH/0155).

## Results

See Tables 2 and 3 for participants and data collected.

**Table 2:** Data collected

Data sources	Hazeldean				Oakdown				Ashgrove			
Interviews												
Rounds of data collection	1	2	3	4	1	2	3	4	1	2	3	4
CLAHRC leadership role	2	–	–	2	3	–	–	2	4	1	2	2
Boundary spanning/implementation role	9	3	7	2	1	3	4	–	4	5	3	1
Academic	5	–	1	2	4	3	–	3	–	3	1	4
Clinical academic	2	–	–	1	–	1	1	1	2	1	–	–
Clinician	2	2	–	1	–	3	1	–	–	1	–	–
NHS leadership role	–	3	–	–	–	2	–	2	–	–	–	–
PPI role	–	–	–	–	–	–	–	1	–	–	–	1
Within case totals	20	8	8	8	8	12	6	9	10	11	6	8
	44				35				35			
Observation of CLAHRC Board meeting	One meeting (12 participants)				–				–			
Feedback from round 1 data collection to those in leadership roles in CLAHRCS	–				3				2			
Observation data from feedback sessions/workshops with mixed attendees	One session (24 participants)				–				One session (21 participants)			
Sub-total	36				3				23			
Documents	17				6				8			
Total reach	80				38				58

**Table 3:** Participants in interpretive forum

Members of 7 CLAHRCs	15
Academics with an interest in knowledge mobilisation	3
Policy makers	3
Members of the research team	7
Total	28

### Collaboration as a context for implementation

The findings are organised around the context-mechanism-outcome configurations we uncovered and refined over the four rounds of data collected, and which we verified with stakeholders in the interpretive forum (see Table 4 for a summary of the CMOs).

**Table 4:** Summary of CMOs

Conceptual, cognitive and physical positioning of stakeholders at micro, meso and macro levels led to individual, group and CLAHRC interpretations of collaborative action, which resulted in setting and sustaining a particular direction of travel or path dependency, including approach to implementation.
CLAHRCs’ governance arrangements including both structures and processes between people, places, ideology and activity prompted different opportunities for connectivity which impacted on the potential for productive relationships and interactions for collaborative action around implementation.
Positioning and availability of resources, including funding for implementation, roles, opportunities, and tools prompted facilitation resulting in a range of impacts including engagement, capability and capacity building, improved care processes and patient outcomes and personal benefits.
Stakeholder agendas and competing drivers prompted different motivations to engage resulting in a variety of understandings about CLAHRC goals and outcomes.
A CLAHRC’s receptiveness to evaluation and learning led to review and reflection, which results in adaption and refinement.

These CMOs are represented in figures, which show a contingent relationship between contexts (left-hand side) and mechanisms (right-hand side) to result in outcomes.

### Starting point

**Fig. 2:**
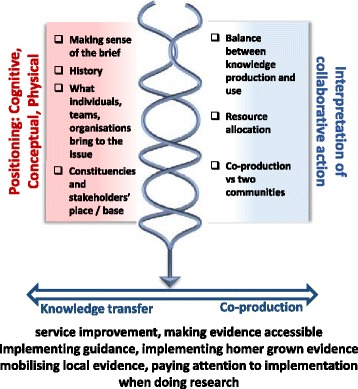
Starting point

There were a number of antecedent conditions which influenced the subsequent course of CLAHRCs. The conceptual, cognitive and physical positioning of stakeholders at micro, meso and macro levels (*context*) led to individual, group and CLAHRC interpretations of collaborative action (*mechanism*), which resulted in both setting and sustaining their direction of travel, including approach to implementation on a knowledge transfer to co-production continuum (*outcome*) (Fig. 2).

#### Positioning and interpretation

The CLAHRCs started from different positions in relation to the nature and quality of existing relationships between constituent higher education institution(s) and health service(s). In Oakdown, pre-existing relationships were in place at the time of bid development and were perceived to provide the ‘intimate fabric there to build on and for people to engage with’ (Leadership role, Oakdown). In contrast, Hazeldean and Ashgrove’s starting positions did not appear to be built on historical organisational connections, which meant relationships were being established on an activity by activity basis:

So it’s really taken 12 months, I think to build these clinical relationships with colleagues in primary care…get themselves known, to be accepted (Clinical academic, Ashgrove)

We observed that more established existing relationships catalysed collaborative contexts in a shorter period of time. The role and function around what the NIHR brief called the ‘application of research findings’ (i.e. implementation) was a combined function of what they brought to the issue (‘conceptual position’) and their interpretation of the brief (‘cognitive position’) specifically:

- Their existing knowledge about, and expertise in, implementation, which some admitted they were on a journey of figuring it out: ‘we just don’t know how to do it…a lot of us just aren’t up to speed with this (implementation) agenda’ (Leadership role, Ashgrove)
- Their interpretation of collaboration between services and academia, and whether knowledge production and use were perceived as a more or less collaborative act. Oakdown, for example, explicitly set out to avoid CLAHRC being perceived as ‘some sort of piglet research programme that has a limited life…’ (NHS senior leadership role, Oakdown). As such, they espoused ‘co-production’ as their preferred approach and ‘to provide a framework which will make it more systematic to think about how you scope evidence and apply it in practice when you are working on a particular issue’ (Academic, Oakdown).

Physical proximity was both a condition for and a function of how CLAHRC leaders viewed implementation as a collaborative act/process, for example, whether the executive team was situated within health services or a higher education institution. Being physically embedded in the service context was symbolically important in the context of the *raison d’être* of a CLAHRC.

Whilst there was significant potential for a flexible interpretation of the NIHR call, the position of those writing the bid triggered their reading of it—particularly in relation to the balance between their role as producers and users of applied health research. As these interpretations were fixed into the contract with the funder, they played out in a pervasive way because they were evaluated against what they proposed to deliver. There had been a trade off in focus and therefore resources (financial and human) between conducting research and undertaking implementation-related activity. Overall across all three CLAHRCs, the balance tended to be weighted more towards the generation of evidence than its application:

I suppose 80 % more or less for applied health research themes and one implementation theme, broadly the money’s been distributed 20 % each…I think if I were bidding again we could certainly change that distribution (Leadership role, Ashgrove).

However, there was an expressed aspiration that both the creation and use of knowledge in practice could be achieved through co-productive ways of working, which was most evident in Oakdown. Facilitating this aspiration closer to reality was dependent on the quality of working relationships and through establishing structures/activities that facilitated more integrated ways of working between the two communities of service and higher education (described in proceeding sections).

#### Implementation continuum

The different positions and interpretations came together to result in a mixed picture of implementation. As highlighted above, the balance of activity was weighted towards research production rather than its use in practice; however, we were able to distil a number of approaches to mobilising knowledge:

- *Service improvement*—i.e. implementation of quality improvement methods to improve specific service and/or clinical issues—e.g. helping GP practices to make an earlier diagnosis for patients with particular conditions (evident in outcome data from all three CLAHRCs)
- *Making evidence accessible*—i.e. converting evidence/guidance into more practical and potentially useable products, e.g. taking blood pressure targets and developing aide-memoirs for clinical staff (evident in all three CLAHRCs related to their relative focus on implementation activity)
- *Taking national evidence and getting it into practice*—typically evident through a focus on the implementation of guidance into local services, e.g. improving venous thromboembolism (VTE) care based on national guidance and quality standards through local facilitation of a VTE assessment tool
- *Mobilising local evidence*—i.e. sharing intelligence about local evidence of effective practice within and across CLAHRCs, e.g. using knowledge from one improvement project to inform a different initiative (more evident in Hazeldean and Oakdown)
- *Paying attention to aspects of implementation in the conduct of research*—for example, within trials of clinical interventions, paying attention to implementation processes in addition to clinical effectiveness outcomes (particularly evident in Ashgrove)
- *Using home grown evidence*—a funder expectation, which largely remained an aspiration given the initial funding period, e.g. Ashgrove implemented their research through incorporating it into an online tool

This categorisation could be placed on a knowledge transfer to co-productive continuum, with the overall balance being weighted towards knowledge transfer-type approaches (rather than co-production). This outcome makes visible the particular conceptual, cognitive and physical positioning resulting in an interpretation of evidence use as something slightly removed or separate from the service, which reinforced a ‘potential disconnect between the priorities of the NHS and the work that is being done [in the CLAHRC]’ (Leadership role, Ashgrove).

### Connectivity

**Fig. 3:**
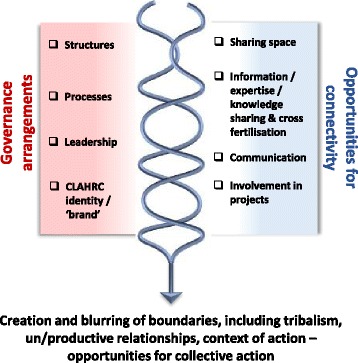
Connectivity

As a distributed model for the conduct and application of applied health research across a wide regional geography, a number of features of a CLAHRC’s organisation or architectures influenced communication, collaboration and potential for collective action on implementation. Their engineered (structure), aesthetic (brand/identity) and social (culture) architectures created governance arrangements (*context*) that prompted varying opportunities for connectivity (*mechanism*), which impacted on the potential for productive relationships, interactions for collaboration action on implementation and the development and maintenance of boundaries (*outcome*) (Fig. 3).

#### Governance and connectivity

The degree of fit and integration across different activities and constituencies varied with some being more joined up than others. Engineering the work of the CLAHRCs within themes and functions (implementation, research, clinical etc.) had been counter-productive to working in an integrated way: ‘it doesn’t work in parallel…because they…went off and did their own thing it meant strands went in different directions’ (Academic, Hazeldean). In all three CLAHRCs, the structures were not obviously connected, although Oakdown’s internal reporting structures and processes had enabled some information sharing:

…a lot of things around the governance and reporting structures actually helps to build that culture of what is expected and to consolidate that transfer… individual projects without that organisation would be lost. (Leader, Oakdown)

The social architects of CLAHRCs were their leaders: ‘the leads very much feel they have influenced the thinking behind the CLAHRC and how that CLAHRC has evolved over time’ (Leader, Oakdown). They shaped the environment and set the tone that encouraged behaviour and action towards certain goals, i.e. evidence use versus research production, which linked back to their position on the interpretation of the brief and the creation of a more or less facilitative milieu for internal and external stakeholders to engage with. A more centralised or top-down leadership approach such as that seen in Hazeldean led to closer and tighter networks that were more difficult for people to penetrate:

…it was quite a traditional leadership style …so it was much easier to keep people in their clearly defined boxes and manage those is a conventional vertical way rather than risk putting people together in much more informal cross projects, cross functional groups and my sense would be that is probably where some of the barriers developed…there was relatively little horizontal movement of people or information or knowledge because the systems weren’t set up in that way really*.* (Leader, Hazeldean)

This contrasted with leadership that was more distributed across the CLAHRC through informal leadership, for example in Oakdown and Ashgrove, which provided opportunities for engagement at various levels. More centralised leadership was also described as ‘command and control’ and ‘divide and rule’ in contrast to those encouraging a more distributed approach perceived as ‘open and facilitative’. These different styles provoked responses that were practical, for example, ‘keeping people in their clearly defined boxes’ (Leader, Hazeldean), and emotional, ‘I found the whole thing (being involved in a CLAHRC) challenging’ (Manager, Hazeldean).

CLAHRC itself was not a well understood concept, particularly for service providers who held a perception of it being an ‘academic machine’. The challenges with the branding of CLAHRC resulted in the need to actively sell the benefits and opportunities of getting involved. Those working in boundary spanning roles were particularly important in prompting connections through their interactions and activities with both academics and practitioners:

And so establishing and doing these sort of teaching sessions at first, we did quite a lot of study days where staff came from the Trust. And [name of facilitator] was the biggest help in getting over any sort of barriers and boundaries because she was there, she knew the Trust, she could sort of go and…work with the staff at a ward level…and constantly reinforce the message. (Boundary spanner, Oakdown)

#### Boundaries and collective action

The structures of the CLAHRCs had in some cases emphasised the professional and epistemic differences between higher education and practice because they reinforced both the metaphorical and physical distance between them:

There was quite a lot of siloed behaviour of that being research and that being implementation (Clinical Leader, Hazeldean)

This also played out in behaviour through tribalism between groups:

if you helicoptered over this room…it had all the implementation theme down one end of the room…and then there was the research team up one end (Leader, Hazeldean).

The way in which these CLAHRCs had engineered their architectures resulted in boundaries between research and practice, higher education and health services. The different perspectives individuals and groups brought to the issue perpetuated professional and epistemic boundaries and these resulted in semantic boundaries. The geographic delineation of the CLAHRC and network of CLAHRCs resulted in physical and spatial boundaries. Table 5 summarises the types of boundaries observed as a result of the interaction between governance arrangements and opportunities for connectivity.

**Table 5:** Types of boundaries

Type of boundary	Nature of the boundary
Organisational	Between different organisations and divisions/departments within and across institutions
Epistemic	Between the different philosophical perspectives individuals, teams and organisations have about knowledge, its provenance and its mobilisation
Semantic	Between people and groups because of different understandings about meaning and language
Professional	Between different professional groups in different contexts
Geographic	Between the CLAHRC network (of nine) and within CLAHRCs (and their constituencies)

These conditions reduced the opportunity for interaction, communication and working together in general, and specifically, in implementation-related activity. Despite the call for collaboration embodied in the CLAHRC concept, in practice, participants’ reflections often represented different points along a co-operation to collaboration continuum. Less integrated structures were overcome by creating opportunities and space for connections to be made between services and higher education and for ideas and knowledge to be shared amongst different communities (e.g. events, learning opportunities, projects). The added value of these opportunities was that they made the CLAHRC more visible and increased the potential of individuals feeling connected to ‘a’ CLAHRC. This had led to the development of some productive working relationships particularly at the level of projects, and specifically through service improvement type initiatives, which had also resulted in positive health benefits for patients, for example in earlier assessment and detection of a particular disease across a region (in Hazeldean).

### Spanning boundaries

**Fig. 4:**
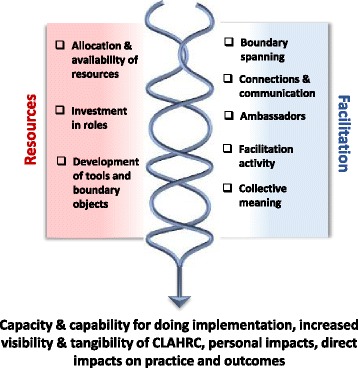
Spanning boundaries

Facilitative capacity and capability was affected by the CLAHRCs’ approach to implementation and the associated resources consequently available for implementation. This focus and resource allocation reinforced a persistent direction of travel or path dependency described above in positioning and interpretation. The positioning and availability of resources, including funding for roles, opportunities and tools (*context*), prompted facilitative potential (*mechanism*) resulting in a range of impacts including engagement, capability and capacity building, improved care processes and patient outcomes and some personal benefits for role holders (*outcome*) The interaction between resources and facilitation (expressed as enabling, freeing up, helping and making things easier) catalysed/stimulated the potential for action (Fig. 4).

#### Resources and facilitation

The allocation of resources for the creation of formal boundary spanning and facilitation roles was the CLAHRCs’ most visible investment in implementation and collaboration building activities:

I think what has become evident for us is the importance of boundary spanning at different levels… it is about the importance of boundary spanning at executive and Board level going down to middle managers and steering committee members, key clinicians in the Trust as well as middle managers and then importantly boundary spanning with frontline staff, the people whose practice we are trying to change… (Leader, Oakdown)

Knowledge broker roles between services and academia were most evident in CLAHRCs where the balance was weighted towards research production (than use) and where knowledge transfer was the dominant model of implementation: ‘….we needed something to bring the organisations and researchers together…’ (Lead, Ashgrove). In contrast, facilitators (those engaged in implementation activity) were more visible where there was more of a focus on projects and activities: ‘to help facilitate that change in practice’ (Lead, Oakdown), for example in guidance implementation and improvement projects.

These individuals were important ambassadors for CLAHRCs; they were its visible face and as such made ‘it’ a more tangible entity. Their personal qualities and skills, and consequent credibility, were important conditions for success in their roles. For those in healthcare organisations, their response to the presence and work of these individuals was enhanced by perceptions of their credibility which facilitated boundary spanning:

I think the [facilitators] have done a really good job of bridging the boundaries between the University and the NHS, not perfectly, but at a relationship level they have (Leadership role, Hazeldean).

The availability and targeting of resources in implementation and improvement projects and in the development of tools released facilitative potential in that they created opportunities for cross boundary working, communication and connections. The collective generation of clinically relevant tools (for example, a venous thromboembolism assessment form or a chronic kidney disease improvement guide) resulted in their use, which had impacted on care processes and outcomes. As such, these tools acted as boundary objects, i.e. they had the potential to facilitate meaning and common understanding between individuals and/or groups. The potential of artefacts such as tools and resources to develop as boundary objects was a function of their collective generation, amendment and tailoring, which provided opportunities for stakeholders to attach meaning to them and enhanced the potential for them to be valued and used.

#### Accumulation of impacts

Over time, positioning and availability of resources, and facilitative capacity and capability led to an accumulation of impacts. This was the foundation upon which further impacts were catalysed and accrued. Accruing impacts was a function of the time needed to establish relationships, priorities, work-plans and then commencement activity. There was evidence of a shift from what could be described as conceptual and processual impacts, including building capability and capacity in the system for ‘doing’ implementation, to those that were more direct—i.e. actual changes to practices and service outcomes. Actual changes to practices were particularly evident in relation to the conduct of improvement projects. As such, Hazeldean and Oakdown who had both used this approach as part of their implementation activity accumulated a number of direct impacts within the funding period (e.g. improvements to outcomes of patients with chronic kidney disease). Across all three CLAHRCs, their investment in boundary spanning type roles had resulted in personal impacts, such as career development and opportunities for building that individual’s personal profile.

### Getting engaged

**Fig. 5:**
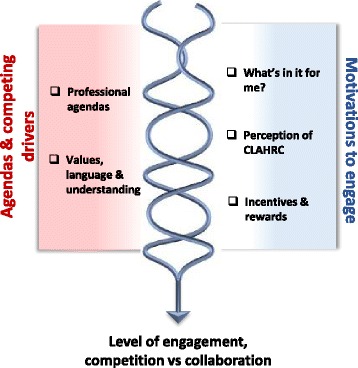
Getting engaged

A CLAHRC is an amalgam of many stakeholders at individual, group and organisational levels, each with different agendas and competing drivers. These stakeholder agendas and competing drivers (*context*) prompted varying motivations to engage and disengage (*mechanism*) resulting in varied understandings (sceptical, unsure) about CLAHRC (as a concept), its goals and outcomes (*outcome*) (Fig. 5).

#### Competing drivers and motivations to engage

The context of competing drivers and different agendas triggered different motivations to engage with the CLAHRC early on in its set up and over time. This was evident across all three CLAHRCs and in the different motivations between service-based individuals, and academics. These motivations were made visible through the views of stakeholders about the purpose of CLAHRCs and who they were there to serve. CLAHRC was viewed as something separate, an external organisation and therefore there were questions about who ‘belongs’ to it, which also linked to how its architecture, including its identity, facilitated or impeded opportunities to connect to it—practically (getting involved in activities) and conceptually (feeling part of it).

One manifestation of the mechanism of motivation was how ‘what’s in it for me’ was enacted through the incentives and rewards that were perceived to be available through CLAHRCs. The academic-practice divide played out strongly as a context for motivation to engage, for example:

.*..*we [NHS] have invested a good deal of money and we want to see ourselves get something out of it…something that will benefit patients (Clinical lead, Ashgrove), in contrast to an academic’s perspective:

I don’t think it worked from a REF (a national research excellence exercise)*…* I would be interested to know how many people involved in CLAHRC have been producing international style papers with T2 style research (Academic, Ashgrove).

In contexts where there was an absence of a history of established relationships and collaboration, where there has been less activity around the joint setting of priorities either at bid development stage, the need to sell the benefits of CLAHRC to encourage or incentivise engagement of mutual benefit was evident:

I think that getting people engaged in it is about them seeing there’s some mutual benefit and where there wasn’t seen as any mutual benefit it doesn’t happen…I know other CLAHRC Directors feel a bit the same, that you feel like you’re a salesperson going round trying to sell things. (Leader, Hazeldean)

Tapping in to the different motivations of stakeholders was a useful mechanism, for example, selling the message to health services that this is what a CLAHRC can do for you to help you meet your CQUINS targets or your service improvement challenges (for example):

The…project and the…project were identified by the organisation because they were CQUINS targets and if they didn’t meet the CQUINS targets they would lose a percentage of their income. (Leader, Oakdown)

There was evidence that this approach did incentivise and catalyse engagement at a project level.

#### Engagement, collaboration and competition

As the above describes, motivations to engage in the context of different agendas and competing drivers resulted in variable levels of engagement along a co-operation to collaboration continuum. This included motivations to work across CLAHRCs which we observed being eroded by the second call for CLAHRCs, which also prompted competition:

Going into the second round of funding applications there was an organisational decision that we wouldn’t share very much about what we had learned and what we were doing and what we were planning…we were in a competitive environment…I think it potentially had a negative effect on the national programme as a whole…it wasn’t an environment that was very conducive to collaboration, and sharing; it was more an environment which was very competitive (Leader, Oakdown)

There was a reinforcing loop in that any exchange, including engagement, needed to result in mutual benefit for the stakeholders (individuals, teams and organisations) involved.

### Learning opportunities

**Fig. 6:**
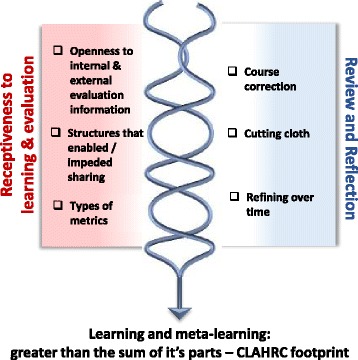
Learning opportunities

The receptiveness and openness of a CLAHRC to evaluation and learning, the way in which they had organised themselves and the types of evaluation and review data that were valued (*context*), prompted opportunities for review and reflection (*mechanism*), which over time resulted in thinking about and doing things differently, and in some learning (*outcome*) (Fig. 6).

#### Receptiveness, review and reflection

The conditions for review and reflection within a CLAHRC were linked to their openness and receptiveness to evaluation, which was a response to the following:

- Leaders’ approaches (i.e. openness to critique and learning)
- The way that they had organised themselves (i.e. themes without communication mechanisms impeded connections and information sharing), and
- The type of evaluation and review information that was valued and therefore collected (i.e. the data that could incentivise review and reflection).

Connections between individuals, groups and projects were facilitated or impeded by the way that a CLAHRC was structured. Whilst there had been some potential for information sharing, there seemed to be fewer opportunities for learning from the information collected and/or shared. Structures and processes to facilitate review and learning within and across CLAHRCs were limited: ‘I don’t know that we’ve done enough on evaluating the whole approach’ (Leader, Hazeldean).

Whilst all CLAHRCs had to collect metrics about their progress for the funder (e.g. grant income, papers published, case studies of impact), this had been potentially restrictive and resulted in less attention to processes and other types of impact:

*…*NIHR are one of the stakeholders…who put a particular performance management framework on what you do…you have to make a decision…whether you allow that to drive the design of your CLAHRC, which is what we did…I don’t really think if CLAHRCs are going to achieve what they need to achieve with their partners, you can do that (Leader, Hazeldean)

The original course of two of the CLAHRCs we studied had been disrupted by events that required responsive action. For example, the course correction taken by one CLAHRC following a review of activity demonstrated their openness to do things differently and reallocate both attention and resources to implementation. Another CLAHRC had to cut their cloth because of changes in the NHS landscape, which resulted in the reduction of financial resources into the programme and a radical adaptation of original plans. We observed a more incremental approach in the other CLAHRC where there was evidence of reflection on how things might be done differently, particularly the way they had organised their activities (e.g. fewer divisions between structures).

#### Learning, meta-learning: the CLAHRC footprint

Learning within and across the CLAHRCs was patchy with varying levels of receptiveness and therefore attention being given to evaluation, and structures and processes that could mediate feedback and learning, for example:

I don’t think had a good enough internal evaluation strategy…so I don’t feel that we have pulled all the learning we have done in a systematic a way as possible. I think looking at the individual [name of] project in the CLAHRC programme, the communication across the projects could have been much, much better, it has ended up almost feeling like they are in competition to each other rather than one big implementation effort. (Leader, Hazeldean)

As this quotation indicates, learning at a project level was more evident than learning from the CLAHRC as a whole; however, from our data, it was not clear how project learning was being incorporated into doing things differently as they progressed during this initial funded period. It was evident, however, that learning from the CLAHRC ‘pilot’ had been taken forward into the proposals in response to the continuation of CLAHRCs and renewed funding (e.g. building in more opportunities for sharing and learning). Participants at the interpretive forum reflected on not formally evaluating the question ‘what have we learnt’, which was expressed as a missed opportunity. The potential to scale up from a set of activities or from a group of projects and for meta-learning was not realised in the data we gathered from the CLAHRCs we studied. As such, their regional footprint was not yet clearly visible.

## Discussion

In contrast to the majority of the evidence base (with some notable exceptions, e.g. [31, 32]), we studied collaboration-based implementation from a longitudinal perspective and presented a temporal narrative explanation for the potential of CLAHRCs or other models of organisational collaboration to close the ‘gap’ between research and practice.

As might be expected, these collaborations developed and evolved over time, which started with their position on collaboration, knowledge and implementation. These positions influenced how implementation within the context of collaboration was organised and operationalised. The degree of alignment between these positions and other features determined outcomes, including the potential to create shared spaces for collective action. The interplay between starting position, organisation, operationalisation and resultant impacts was influenced by a network of actors, including boundary spanners. These issues are discussed more fully below.

### Implementation as a function of a collaborative life cycle

CLAHRCs did not emerge from a ‘vacuum’. Being able to track the journey of three collaborations over time led us to identify the potential of a life cycle as they evolved and developed. The life cycle of the CLARHC and more fundamentally the stage of development of the collaboration between the constituent partners provided the conditions for any potential collective action around implementation. Our findings indicate that CLAHRC-like entities need time to learn and develop [13], particularly in the absence of a shared history [11, 33], or any pre-formative work [34–36].

CLAHRCs are complex, in the sense that their behaviour can be explained with reference to the properties of a whole (adaptive) system rather than its individual components. This complexity theory lens views outcomes emerging from interactions amongst individuals within a system through an evolutionary and emergent process of self-organisation [37, 38]. This reflects the CLAHRCs’ formation and development, which was iterative and emergent as individuals and groups within the collaboration went through a process of sense-making, a finding also supported by others’ research into CLARHCs [39]. A history of working together catalysed collective action (and therefore, impacts) in a shorter time frame [40]. The evolution of a CLAHRC could, conceivably, be made more efficient by ensuring that learning and process and outcome feedback are designed into the collaboration’s structures and activity.

### Alignment

Out findings show that within these types of collaborations, ‘things coming together’ provided the conditions for impacts to be achieved. As Jagosh et al. [41] remind us, collaborations are not de facto synergistic but predicated on some effort to align values, goals and purpose. More impactful attempts at collective action in implementation were determined by the deliberate alignment of a number of features, including foundational relationships, vision, values, structures and processes (including the potential for meta learning), purpose and thoughts about the nature of the collaboration *and* implementation (including relevant theory and tried-and-tested approaches). Leadership (designated and distributed, style and approach) were a critical part of CLAHRC’s governance arrangements and therefore to establishing the collaboration and to determining and then enacting a vision around implementation. Alignment between formal leadership and distributed or shared leadership functions can reduce ‘cognitive dissonance’ and facilitate integration between intra-organisational boundaries [42]. Further, where structures, positions and resources were aligned, this released the potential and unlocked barriers for purposeful collective action, the successful delivery of projects and the potential for positive impacts on processes and outcomes. We suggest that distributed leadership, coupled with a shared vision and influence might be the ‘oil that helps lubricate the system’.

Within an ecological view of implementation, synergy has the potential to build and develop over time, with impacts from collaborative implementation activity providing reinforcement. Additionally, alignment has the potential to develop over time where there is attention to learning and evaluation with appropriate adaptation [41]. However, some tension within the implementation ‘system’ could also potentially act as a form of catalyst for action and minimise the potential for entrenchment and habit.

### Shared space

Collaboration had the potential to provide the structure and opportunity for developing shared space(s) in and around which implementation could occur. The extent to which this sharing occurred was dependent on each CLAHRC’s position on the knowledge transfer-co-production continuum. However, Orr and Bennett point out that ‘tricky issues’ arise from ‘co-producing research involving cooperative interactions between members of two communities that have distinct interests, expectations and priorities’ [43]. Relinquishing influence and power to achieve genuinely shared space is notoriously challenging [44].

Negotiation and re-negotiation of shared physical and cognitive space in the development of collective action was explained by interactions between people and multiple contexts:

- *Temporal*: historical, longitudinal and living/emergent history of relationships and working together
- *Cognitive*: collective and individual notions and epistemology about collaboration, evidence, knowledge and implementation,
- *Emotional*: thoughts and feelings of individuals and the collective: what people thought about the CLAHRC, collaboration and how they enacted ‘what’s in it for me’
- *Professional*: disciplinary silos/professional ‘tribes’, power, language
- *Physical context*: governance, structures, processes, physical and social geography
- *Political context*: reward and incentive structures/frameworks (e.g. REF, funder expectations), organisational/service changes/redesigns

These contexts were not a backdrop to action [45], but rather they coalesced to create the conditions and contingencies that explained whether there was potential to provide opportunities for connectivity and engagement and to develop genuinely shared space(s) for collaborative action around implementation.

### Networks of actors

CLAHRCs are networks of stakeholders and have variously been described as a ‘constellations of inter-connected practices’ [46, 47] and communities of practice [48]. The opportunities for connectedness and connectivity within and across disparate networks of actors were influenced by CLAHRCs’ architectures. Within this networked model of collaboration, we noted the creation and negotiation of boundaries. Where boundaries were negotiated and knowledge sharing and/or implementation occurred, this was as a result of the creation of boundary objects and through the agency of those in boundary spanning roles.

Bridging, brokering and boundary spanning roles have a key role in cross fertilisation of ideas between groups, for generating new ideas and for increasing understanding and cooperation [49, 50]. Our findings showed that individuals in boundary spanning roles managed implementation activity/projects (i.e. were facilitators of evidence in to practice), facilitated interactions into productive conversations and action, helped develop shared spaces and negotiated tensions. Others have stressed therefore the importance of the position of people in these roles within appropriate networks [51]. These roles and individuals were an essential CLAHRC component, and we suggest that without them, the quality of interaction (and de facto, collaboration), and implementation impacts would have been limited.

Tools in the armoury of boundary spanners included their human capital, i.e. their advantage in terms of personal attributes including credibility and skills. They also embraced opportunities to develop ‘artefacts’ (e.g. disease register, alert card) that had the potential to become boundary objects. We found that what activated or catalysed an object to become something that people from different territories crossing various boundaries could attach meaning, resonance and value to [52], was related to how they evolved. Those artefacts that transformed from objects without meaning to boundary objects shared generation through collective action. The opportunity for meaningful collaboration provided relevant stakeholders to come together and engage in a process that involved integrating local evidence, experience with external evidence from guidance. The collective design process made the artefacts meaningful and contextually situated—i.e. made them boundary objects, which led them to be used in practice. As such it could be hypothesised that where there are planned opportunities for co-design and creation involving relevant stakeholders, the catalytic properties of potential boundary objects could be enhanced.

### Summary

**Fig. 7:**
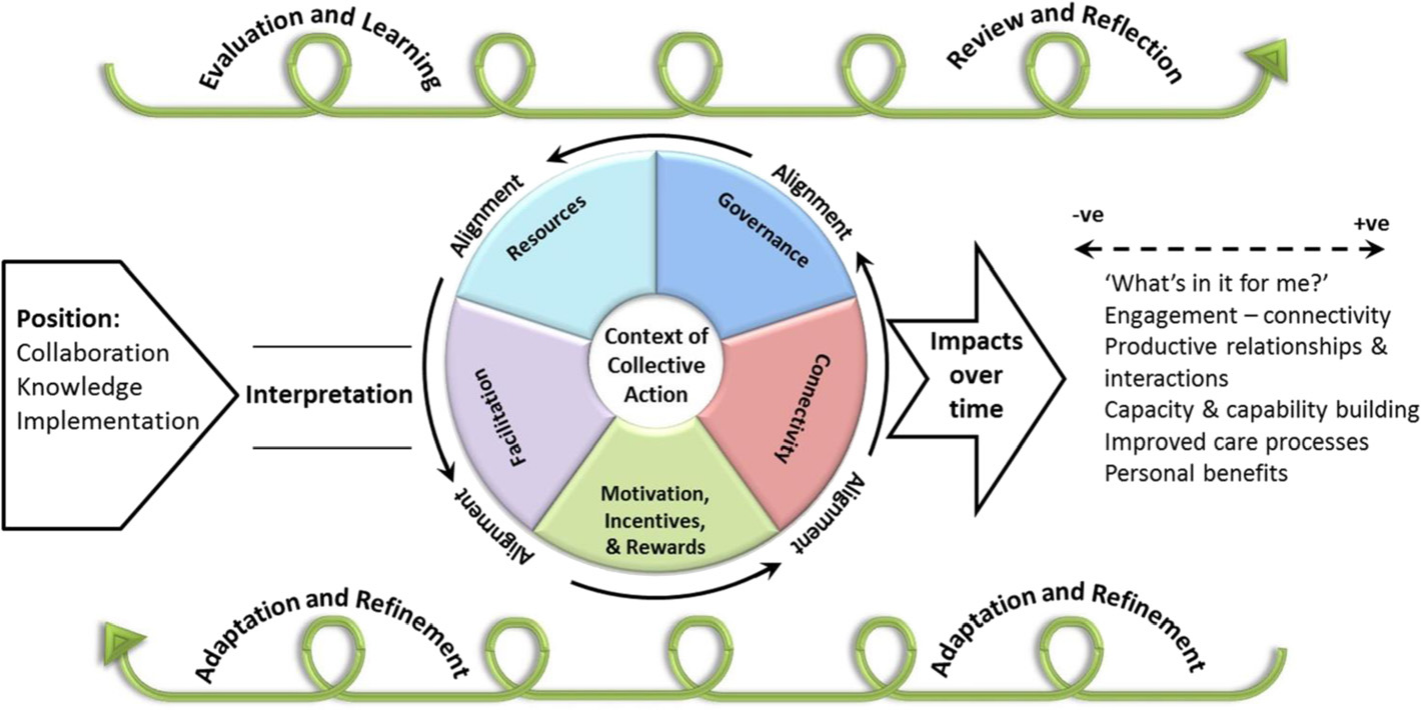
Representation of contingencies between CMO configurations

The interconnections between these CMOs create the potential for collective action in implementation, which is represented in Fig. 7. Figure 7 shows a path dependency, which starts with the position of stakeholders on the key issues of collaboration, knowledge and implementation. Collectively, these positions influence how implementation within the context of collaboration is both organised and operationalised. The degree of alignment between these positions and features determine outcomes. We hypothesise that greater alignment leads to impacts that are more timely and relevant for stakeholders and services. The development and progress of implementation through collective action will be influenced by the collaboration’s approach to evaluation and learning and their subsequent response to triggers or events.

### Limitations

We studied three CLAHRC in detail and therefore cannot make claims about how our findings are representative of other CLAHRCs. However, we have provided description to enable readers to make a judgement about the theoretical transferability of the findings to different contexts [30]. Some degree of face validity/member checking (and therefore trustworthiness) was afforded to our findings by having CLAHRC participants themselves on our team. Such methods are comparatively uncommon.

Our approach to sampling may have introduced some bias (e.g. availability, recall or social desirability), which we attempted to minimise through triangulation of data sources and establishing trustworthiness of emerging findings in feedback sessions with a wider group of participants, e.g. in the interpretive forum.

## Conclusions

In this study we had the opportunity to trace the journey of three large-scale organisational collaborations grappling with knowledge production and implementation in the context of cultivating meaningful and productive relationships with multiple partners. Our findings have the potential to provide a reusable conceptual platform [25] about implementation within the context of organisational collaboration. Within this explanation, we propose the following middle range theory for collective action for implementation.

- If working relationships are already established or there has been some pre-formative work, then this is more likely to lead to quicker wins and a greater appreciation of each other’s positions and purpose upon which to build plans and activities.
- If there is a lack of attention to evaluation/learning, and leadership teams are not reflective, then the initial interpretation of the ‘brief’ will create a path dependency that is difficult to alter, particularly if it is reinforced by funders’ expectations. Therefore, building in mechanisms for evaluation, learning and meta-learning within and across projects/programmes/activities/the collaboration, which feeds into adaption and refinement of implementation plans will facilitate resilient collaborations and sustainable activity particularly in contexts that are in constant flux (i.e. health services).
- If the governance framework for the collaboration, including its architectures and processes/activities, facilitates opportunities for physical (and face to face), social and intellectual connectivity between stakeholders, then this is more likely to lead to productive conversations, the shaping of more conducive contexts of action (temporal, cognitive, emotional, professional, physical and political), and implementation and/or co-productive activity that resonates with participants.
- If there is a shared vision and some alignment across stakeholders’ views about, and position on knowledge production and use as a collaborative act, which is also aligned with the collaboration’s governance framework, including targeting of resources then this is more likely to lead to unblocking barriers for purposeful collective action.
- If the motivations for engagement (‘what’s in it for me’) are made visible within and across individuals, professions and organisations then implementation activity can be planned so that engagement in it is appropriately incentivised.
- If resources are invested in boundary spanning mechanisms, such as in credible and appropriately prepared individuals working in brokering and facilitation roles, and in opportunities to develop tools or artefacts in a way that have the potential to acquire the properties of boundary objects, then this will lead to bridging boundaries, and in catalysing implementation activity.
- If there is some tension in the system between collaboration and competition then this can be facilitative, as well as an inhibitory force—therefore, finding and monitoring the balance is an important function of leadership.
- If there is strong (clear vision, thoughtful/strategic allocation of resources, reflective, visible) core leadership combined with distributed leadership (e.g. through boundary spanners) then this will facilitate collaboration, and subsequently the potential for collection action around implementation.

Our explanation captures the interactive nature of users and producers of knowledge brought closer together in order to generate and implement applied health research. Table 6 translates this explanatory theory into some action statements.

**Table 6:** Action statements

• Identify opportunities for quick wins that build on earlier or pre-formative collaborative work and/or dialogue.
• Ensure there are opportunities for learning and evaluation and that these can feed into changes in ways of working around implementation.
• Create a flexible architecture and clear processes for ways of working across the partnership(s), which allow interaction and productive conversations.
• Check out stakeholders understandings of implementation, and build (interactively and iteratively) a middle-ground for collective action.
• Use incentives to drive engagement that reflect the relevant professional and research contexts.
• Build on existing relationships and networks within and across partner organisations.
• Ensure that facilitation resources (including potential for developing artefacts and tools) and skills are situated where required to catalyse implementation activity.
• Create an integrated mix of formal and distributed leadership around both collaboration and implementation.
• Assume the contexts for collaboration(s) and implementation will change over time, and that there is structural and financial agility to accommodate this.
• Use financial resources and flows across the collaboration(s) to renegotiate, rather than create barriers to collective action on implementation.

In the context of increasing calls for co-production to deal with some of the ‘wicked’ challenges that health and other public services face, our findings highlight that it is not a panacea, and certainly not a quick fix. Furthermore and as a caution, co-production and collaboration could lead to a more complex context that supports additional emergent, unexpected, unintended consequences—and thereby, new ‘wicked problems’.
